# Four new diterpenoids from *Isodon eriocalyx* var. *laxiflora*

**DOI:** 10.1007/s13659-013-0057-0

**Published:** 2013-08-13

**Authors:** Wei-Guang Wang, Xue Du, Xiao-Nian Li, Bing-Chao Yan, Min Zhou, Hai-Yan Wu, Rui Zhan, Ke Dong, Jian-Xin Pu, Han-Dong Sun

**Affiliations:** 1 State Key Laboratory of Phytochemistry and Plant Resources in West China, Kunming Institute of Botany, Chinese Academy of Sciences, Kunming, 650201 China; 2 University of Chinese Academy of Sciences, Beijing, 100049 China

**Keywords:** *Isodon eriocalyx* var. *laxiflora*, laxiflorins, diterpenoid, *ent*-kaurane, *ent*-abietane

## Abstract

Four new diterpenoids, laxiflorins S-V (**1–4**), bearing four different types, and one known compound, laxiflorin O (**5**), were isolated from *Isodon eriocalyx* var. *laxiflora*. Compound **1** was the first example of *ent*-kauranoids bearing a unique C_24_ carbon framework and compound **4** was the first example of 3,4-*seco-ent*-abietane diterpenoids from the *Isodon* genus. Their structures were determined by spectroscopic methods (UV, IR, MS, NMR).

**Electronic Supplementary Material:**

Supplementary material is available for this article at 10.1007/s13659-013-0057-0 and is accessible for authorized users.

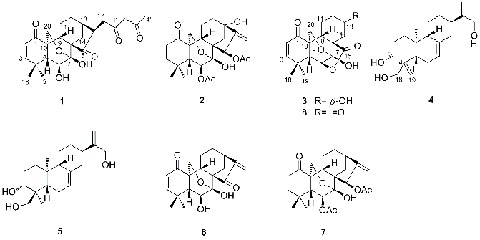

## Introduction

The Natural Products Library, is one of the primary sources for uncovering novel drug candidates, and is highly regarded in drug discovery.[1–3] The construction of an *ent*-kaurane diterpenoids library was initiated by our group in 1976, and more than 1000 pure *ent*-kauranoids, which includes more than 700 novel discoveries, have been identified from *Isodon* genus.[4,5]

As one of the important plants of *Isodon* genus, *I. eriocalyx* var. *laxiflora* has been investigated phytochemically as it is a rich source of diterpenoids, such as 7,20-epoxy-*ent*-kauranoids (laxiflorins H and I),[6] 3,20-epoxy-*ent*-kauranoids (laxiflorins J–M),[7,8] 6,7-*seco*-*ent*-kauranoids (laxiflorins A–C),[9] 6,7:8,15-*seco*-*ent*-kauranoids (laxiflorins F and G),[10] *ent*-abietanoids (laxiflorin N),[11] 15,16-*seco*-16,17-dinor-*ent*-kauranoids (neolaxiflorins D–F)[12], two unprecedented epimeric bishomoditerpene lactones with a unique $C_{22}$ framework (laxiflorolides A and B),[13] and two unprecedented *ent*-kaurane diterpenoids (neolaxiflorins A and B).[14] Our further investigation of this plant led to the isolation of four novel ones, laxiflorins S–V (**1**–**4**), and one known compound, laxiflorin O (**5**)[15] (Figure 1). These new compounds could be classified into 4 different types as below: tetrahomo-7,20-epoxy-*ent*-kauranoid (**1**), 7,20-epoxy-*ent*-kauranoid (**2**), 15,16-*seco*-16,17-dinor-*ent*-kauranoid (**3**), and 3,4-*seco*-*ent*-abietane diterpenoid (**4**), respectively. In addition, compound **1** was the first example of *ent*-kauranoids bearing a unique $C_{24}$ carbon framework; compound **3** was the second 15,16-*seco*-16,17-dinor-*ent*-kauranoid obtained from the *Isodon* genus; compound **4** was the first example of 3,4-*seco*-*ent*-abietane diterpenoid obtained from the *Isodon* genus plants. In this paper, we report the isolation and structure elucidation of compounds **1**–**5**.

## Results and Discussion

Laxiflorin S (**1**) was obtained as an amorphous powder. The molecular formula $C_{24}H_{30}O_7$, has ten degrees of unsaturation, was established based on HREIMS ($[M]^+$, 430.2003; calcd for $C_{24}H_{30}O_7$, 430.1992) and NMR spectroscopy (Tables 1 and 2). The IR spectrum of **1** indicated the presence of hydroxy groups (3480 $cm^{-1}$) and two types of carbonyl group for saturated ketone (1741 $cm^{-1}$), and conjugated ketone (1648 $cm^{-1}$), respectively.

The $^1H$ NMR spectrum (Tables 1 and 2) showed resonances attributed to an $\alpha,\beta$-unsaturated ketone moiety $\delta_H$ 5.70 (1H, ABd, $J$ = 10.1 Hz, 6.41, 1H, ABd, $J$ = 10.1 Hz), two tertiary methyls at $\delta_H$ 1.08 (3H, s) and $\delta_H$ 0.80 (3H, s), and quaternary methyl at $\delta_H$ 1.75 (3H, s). In addition, the spectrum showed resonances due to a characteristic AB methylene group at ($\delta_H$ 4.21 and 3.82, each 1H, d, $J$ = 9.8 Hz), together with an oxygenated methine at $\delta_H$ 3.97 (dd, $J$ = 11.3, 7.4 Hz). The analysis of the $^{13}C$ NMR and DEPT spectra (Table 2) revealed the presence of 24 carbons, which were assigned as three methyls ($\delta_C$ 29.9, $\delta_C$ 24.1, and $\delta_C$ 29.7), six methylenes (one oxygenated at $\delta_C$ 65.6), seven methines (one oxygenated at $\delta_C$ 73.5 and two olefinic groups at $\delta_C$ 127.4 and $\delta_C$ 160.7), and eight quaternary carbons (one hemiacetal at $\delta_C$ 96.3 and four carbonyls at $\delta_C$ 197.4, $\delta_C$ 225.2, $\delta_C$ 207.5, and $\delta_C$ 206.1), which suggested that **1** is a highly oxygenated diterpenoid with a $C_{24}$ skeleton similar to the *ent*-kaurane skeletons previously reported.[4,9] Comparison of the NMR spectral data of **1** with those of eriocalyxin B (**6**)[16] revealed that **1** was consistent with

*To whom correspondence should be addressed. E-mail: pujianxin@mail.kib.ac.cn (J.X. Pu); hdsun@mail.kib.ac.cn (H.D. Sun)

**Figure 1.** Chemical structures of compounds **1**–**8**

a 7,20-epoxy-*ent*-kaurane similar to **6** by reducing the carbon-carbon double bond between C-16 and C-17 of **6** and then adding a butyl-1,3-dione group at C-17.

The HMBC spectrum of **1** showed obvious correlations from the geminal methyls Me-18 and Me-19 to C-3, C-4, and C-5, and from Me-4' to C-2' and C-3'. In addition, the AB methylene $H_2$-20 showed HMBC correlations with C-1, C-5, C-7, C-9, and C-10 respectively. Other HMBC correlations were noted between the AB methine H-2 and C-1, C-3, C-4, and C-10, and between H-3 and C-1, C-4, C-5, C-18, and C-19, between H-6 ($\delta_H$ 3.97) and C-4, C-5, C-7, C-8, and C-10, and between H-17 and C-13, C-15, C-16, C-1', and C-2'. These observed HMBC correlations coupled with three spin systems (i.e., (CHCH, H-2/H-3), (CHCH, H-5/H-6), and ($CHCH_2CH_2CH(CHCH_2)CH_2$, H-9/$H_2$-11/$H_2$-12/H-13(/H-16/$H_2$-17))/$H_2$-14)) established by $^1$H-$^1$H COSY correlations and HSQC spectra suggested the gross structure of compound **1** (Figure 2).

In the ROESY spectrum of compound **1**, the NOE correlations for Me-19/H-6, H-6/$H_2$-20, H-20a/H-14a, H-13/H-16, and H-16/$H_2$-14 suggested that H-6, H-13, C-14, H-16, Me-19, and C-20 adopted the same *α*-orientation. The cross-peaks observed between H-5/H-9, H-5/Me-18, and H-9/$H_2$-17 in the ROESY spectrum demonstrated that H-5, H-9, C-17, and Me-18 all possessed the same *β*-orientation. (Figure 2) Therefore, compound 2 was 6*β*,7*β*-dihydroxy-17-(butyl 1',3'-dione)- 7*α*,20-epoxy-*ent*-kaur-2-en-1,15-dione, and named laxiflorin S.

The HREIMS (*m*/*z* 448.2087, $[M]^+$; calcd for $C_{24}H_{32}O_8$, 448.2097) of compound **2** suggested a molecular formula of $C_{24}H_{32}O_8$, with nine degrees of unsaturation. The $^1$H and $^{13}$C NMR data of **2** (Tables 1 and 2) were consistent with a 7,20-epoxy-*ent*-kaurane, similar to 7*β*-hydroxy-6*β*,15*β*-diacetoxy-7,20-epoxy-*ent*-kaur-16-en-1-one (**7**)[17]. The most notable difference is that the C-13 methine group in **7** was substituted by a hydroxy group in **2**. This difference was supported by HMBC correlations from H-15 ($\delta_H$ 6.40, s) and $H_2$-17 ($\delta_H$ 5.81, d, $J$ = 2.2 Hz and $\delta_H$ 5.44, d, $J$ = 2.2 Hz) to C-13 ($\delta_C$ 75.4, s). The ROESY cross peaks correlations indicated that compounds **2** and **7** had the same relative configuration. Consequently, compound **2** was 7*β*,13*α*-dihydroxy-6*β*,15*β*-diacetoxy-7,20-epoxy-*ent*-kaur-16-en-1-one, and it was named laxiflorin T.

**Figure 2.** $^1$H-$^1$H COSY (▬), selected HMBC (H→C) and key ROESY ( ↶↷ ) correlations of compound **1**

**Table 1.** $^1$**H NMR data (** $\delta$ **in ppm,** $J$ **in Hz,** $C_5D_5N$**) of compounds 1–4**

| Position | 1[a] | 2[b] | 3[b] | 4[a] |
|---|---|---|---|---|
| 1 | | | | 1.53 (m) |
| 2 | 5.70 (d, 10.1) | 1.90 (m); 1.63 (m) | 5.88 (d, 10.2) | 1.88 (m); 1.82 (m) |
| 3 | 6.41 (d, 10.1) | 2.52 (m); 2.07 (m) | 6.49 (d, 10.2) | 3.82 (br. s) |
| 5 | 1.97 (overlap) | 2.85 (overlap) | 2.51 (br. s) | 2.38 (overlap) |
| 6 | 3.97 (dd, 11.3, 7.4) | 5.72 (d, 9.1) | 4.89 (br. s) | 2.36 (overlap); |
| 7 | | | | 5.37 (br. s) |
| 9 | 1.57 (dd, 12.5, 5.4) | 2.83 (overlap) | 2.37 (overlap) | 2.10 (overlap) |
| 11 | 1.14 (m); 0.96 (m) | 2.20 (m); 1.38 (m) | 2.93 (m); 2.31 (m) | 1.76 (m); 1.25 (m) |
| 12 | 1.96 (overlap); 1.42 (overlap) | 2.81(m); 2.38 (m) | 2.19 (m) | 1.93 (m); 1.28 (m) |
| 13 | 2.13 (overlap) | | 5.30 (m) | 2.10 (overlap) |
| 14 | 2.20 (overlap) | 2.63 (br. d, 12.0); 2.43 (br. d, 12.0) | 2.87 (dd,13.8, 6.0); 2.38 (overlap) | 2.50 (d, 11.5); 2.10 (overlap) |
| 15 | | 6.40 (s) | | |
| 16 | 2.13 (overlap) | | | 4.44 (br. s) |
| 17 | 1.89 (m); 1.42 (overlap) | 5.81 (d, 2.2); 5.44 (d, 2.2) | | 5.47 (br. s); 5.04 (br. s) |
| 18 | 1.08 (s) | 0.95(s) | 1.12 (s) | 4.51 (d, 11.6); 4.40 (d, 11.6) |
| 19 | 0.80 (s) | 0.89 (s) | 1.20 (s) | 5.70 (br. s); 5.14 (br. s) |
| 20 | 4.21 (d, 9.8); 3.82 (d, 9.8) | 4.70 (d, 10.3); 4.19 (d, 10.3) | 4.43 (dd, 9.6, 1.8); 4.30 (dd, 9.6, 1.2) | 0.92 (br. s) |
| 2' | 2.18 (overlap) | | | |
| 4' | 1.75 (s) | | | |
| OAc | | 2.37 (s)/2.13 (s) | | |

[a]Recorded at 500 MHz, [b]Recorded at 600 MHz.

The absolute configuration of neolaxiflorin D (**8**), a 15,16-*seco*-16,17-dinor-*ent*-kaurane diterpenoid isolated from the title plant[12], was confirmed by single-crystal X-ray diffraction using anomalous scattering of $CuK_\alpha$ radiation (CCDC 861510).[18,19] Using HREIMS ($m/z$ 334.1416; calcd for $C_{18}H_{22}O_6$, 334.1416), the molecular formula of laxiflorin U (**3**) was determined to be $C_{18}H_{22}O_6$, indicating eight degrees of unsaturation. Its $^1$H and $^{13}$C NMR data (Tables 1 and 2) indicated that it was structurally very similar to neolaxiflorin D (**8**).[12] The most notable difference was that the C-13 carbonyl group in compound **8** ($\delta_C$ 208.7, s)[12] changed into hydroxy group in compound **3** ($\delta_C$ 63.8, d). This structural assignment was supported by the observed HMBC correlations between H-13 ($\delta_H$ 5.30, m) with C-8, C-11, C-12, and C-14, coupled with three spin systems (i.e., (CHCH, H-2/H-3), (CHCH, H-5/H-6), and ($CHCH_2CH_2CHCH_2$, H-9/$H_2$-11/$H_2$-12/H-13/$H_2$-14)) established by $^1$H-$^1$H COSY correlations and HSQC spectra. Thus compound **3** was 7,20-epoxy-7$\beta$,13$\beta$-dihydroxy-1-oxo-15,16-*seco*-16,17-dinor-*ent*-kaur-2-en-6$\beta$,15-olide, and it was named laxiflorin U.

Laxiflorin O (**5**) was an *ent*-abietane diterpenoid isolated from *I. eriocalyx* var. *laxiflora*, and its structure was confirmed by NMR and single-crystal X-ray diffraction in 2002.[15] The molecular formula of compound **4** was determined to be $C_{20}H_{32}O_3$ based on HREIMS ($m/z$ 320.2343, $[M]^+$; calcd. for $C_{20}H_{32}O_3$, 320.2351). A comparison of its corresponding $^1$H and $^{13}$C NMR data (Tables 1 and 2) with those of laxiflorin O (**5**)[15] indicates that compound **4** was the 3,4-cleavage derivative of **5**. The structural difference between these two compounds are that the oxygenated methine group at C-3 ($\delta_C$ 73.9, d) in **5** changed into a hydroxymethyl in **4** ($\delta_C$ 62.9, t), and saturated quaternary carbon at C-4 ($\delta_C$ 38.3, s) and C-19 ($\delta_C$ 23.5, q) in **5** changed into a double bond at C-4 ($\delta_C$ 153.5, s) and C-19 ($\delta_C$ 110.6, q) in **4**. The HMBC correlations (Figure 3) of $H_2$-18 ($\delta_H$ 4.51, d, $J$ = 11.6 Hz and $\delta_H$ 4.40, d, $J$ = 11.6 Hz) with C-4, C-5, and C-19 and of $H_2$-19 ($\delta_H$ 5.70, br. s and $\delta_H$ 5.14, br. s) with C-4, C-5, and C-18, coupled with $^1$H-$^1$H COSY correlations (Figure 3) (for the observed proton spin systems, H-9/$H_2$-11/$H_2$-12/H-13/$H_2$-14, $H_2$-1/$H_2$-2/$H_2$-3, and H-5/$H_2$-6/H-7) confirmed this result. Based on detailed analysis of ROESY data, the relative configuration of the stereogenic centers in compound **4** were determined to be the same as those in laxiflorin O (**5**). The correlations from Me-20 to H-11$\alpha$; from H-11$\alpha$ to H-13$\alpha$; from H-5 to H-9, and from H-9 to H-11$\beta$ was observed in the ROESY spectrum, and thus H-13 and Me-20 were assumed to be $\alpha$-oriented, and of H-5 and H-9 were assumed to be $\beta$-oriented. Therefore, compound **4** was 3,16,18-trihydroxy-3,4-*seco*-*ent*-abieta-4(19),7(8),15-triene, and it was named as laxiflorin V.

**Table 2.** $^{13}$**C NMR data (** $\delta$ **in ppm,** $C_5D_5N$**) of compounds 1–4**

| pos. | 1[a] | 2[b] | 3[a] | 4[b] |
|---|---|---|---|---|
| 1 | 197.4 s | 213.0 s | 200.4 s | 34.3 t |
| 2 | 127.4 d | 38.8 t | 127.7 d | 28.0 t |
| 3 | 160.7 d | 41.0 t | 158.5 d | 62.9 t |
| 4 | 36.2 s | 33.2 s | 37.2 s | 153.5 s |
| 5 | 59.5 d | 54.9 d | 67.2 d | 44.2 d |
| 6 | 73.5 d | 74.1 d | 81.9 d | 30.7 t |
| 7 | 96.3 s | 96.6 s | 101.3 s | 120.4 d |
| 8 | 46.1 s | 51.8 s | 49.6 s | 138.4 s |
| 9 | 48.6 d | 42.7 d | 45.7 d | 44.6 d |
| 10 | 61.1 s | 49.3 s | 47.8 s | 38.2 s |
| 11 | 19.3 t | 20.2 t | 22.1 t | 26.7 t |
| 12 | 19.4 t | 36.2 t | 34.7 t | 32.3 t |
| 13 | 30.0 d | 75.4 s | 63.8 d | 41.8 d |
| 14 | 28.1 t | 36.8 t | 30.3 t | 41.7 t |
| 15 | 225.2 s | 74.2 d | 181.5 s | 155.6 s |
| 16 | 55.5 d | 161.7 s | | 106.9 t |
| 17 | 20.7 t | 109.9 t | | 64.5 t |
| 18 | 29.9 q | 29.9 q | 32.4 q | 66.5 t |
| 19 | 24.1 q | 23.4 q | 24.6 q | 110.6 t |
| 20 | 65.6 t | 65.3 t | 67.2 t | 17.5 q |
| 1' | 207.5 s | | | |
| 2' | 41.3 t | | | |
| 3' | 206.1 s | | | |
| 4' | 29.7 q | | | |
| OAc | | 171.2 s 22.4 q/ 171.2 s 21.7 q | | |

[a]Recorded at 125 MHz, [b]Recorded at 150 MHz.

In summary, four new diterpenoids belonging to four different types as: tetrahomo-7,20-epoxy-*ent*-kauranoid, 7,20-epoxy-*ent*-kauranoid, 15,16-*seco*-16,17-dinor-*ent*-kauranoid, and 3,4-*seco*-*ent*-abietane diterpenoid, have been isolated from *I. eriocalyx* var. *laxiflora* collected in the south-west of China. Their structures were determined by analyses of 1D and 2D NMR spectroscopic data.

Additionally, compound **1** was the first example of *ent*-kauranoids bearing a unique $C_{24}$ carbon framework and compound **4** was the first example of 3,4-*seco*-*ent*-abietane diterpenoid obtained from the *Isodon* genus plants.

**Figure 3.** $^1$H-$^1$H COSY (▬), selected HMBC (H→C) and key ROESY ( ) correlations of compound **4**

## Experimental Section

**General Experimental Procedures.** Optical rotations were measured with a JASCO DIP-370 digital polarimeter. UV data were obtained on a Shimadzu UV-2401A spectrophotometer. A BioRad FtS-135 spectrophotometer was used for scanning IR spectroscopy with KBr pellets. 1D and 2D NMR spectra were recorded on DRX-500 and DRX-600 spectrometers. Unless otherwise noted, the chemical shifts ($\delta$) are expressed in ppm with respect to the solvent signals. HREIMS was performed on a VG Autospec-3000 spectrometer at 70 eV. Column chromatography was performed with silica gel (100–200 mesh; Qingdao Marine Chemical, Inc., Qingdao, China). PPLC was performed on a Shimadzu LC-8A preparative liquid chromatograph with a Shimadzu PRC-ODS (K) column. The fractions were monitored by TLC, and the spots were visualized by heating silica gel plates sprayed with 8% $H_2SO_4$ in EtOH. All of the solvents including petroleum ether (60–90 °C) were distilled prior to use.

**Plant Material.** The leaves of *Isodon eriocalyx* var. *laxiflora* were collected from Yunnan Province, China in September 2009. Voucher specimens (KIB20080028) were deposited at the State Key Laboratory of Phytochemistry and Plant Resources in West China, Kunming Institute of Botany, Chinese Academy of Sciences and were identified by Prof. Xi-Wen Li.

**Extraction and Isolation.** The air-dried leaves of *Isodon eriocalyx* var. *laxiflora* (10 Kg) were extracted with 70% aqueous $Me_2CO$ (3 × 40 L, 2 days each) at room temperature. The solvent was evaporated *in vacuo* to afford a crude extract, which was suspended in $H_2O$ and then successively extracted with EtOAc and *n*-BuOH. The EtOAc-soluble fraction (600 g) was decolorized on an MCI gel with 90:10 MeOH:$H_2O$ to obtain a yellow gum (427.5 g). The gum was purified by CC on silica gel column with a $CHCl_3$:$Me_2CO$ gradient system consisting of 1:0, 9:1, 8:2, 7:3, 6:4, and 1:1 to yield six main fractions (A–F). Fraction C ($CHCl_3$:$Me_2CO$, 8:2; 30 g) was subjected to repeated chromatography over silica gel ($CHCl_3$:MeOH, from 90:1, 60:1, to 30:1) to yield fractions C1–C3. Fraction C2 ($CHCl_3$:MeOH, 60:1, 15 g) was separated by RP-18 CC (MeOH:$H_2O$, 15:85 to 1:0) to afford C2/1–C2/4. Compound **1** (10 mg) was isolated from fraction C2/3 (500 mg) using PPLC (MeCN:$H_2O$, 35:65, 15 mL/min) to achieve this separation. Fraction C3 ($CHCl_3$: MeOH, 5 g) was eluted with RP-18 CC (MeOH:$H_2O$, 10:90 to 1:0) yielding subfractions C3/1–C3/3. Subfraction C3/1 (1.06 g) was fractionated by repeated CC, first on silica gel column with a gradient elution with $CHCl_3$: isopropyl alcohol (60:1 to 20:1) to yield fractions C3/1/1–C3/1/3. Subsequently, fraction C3/1/1 (250 mg) was purified using RP-18 CC (MeOH:$H_2O$, 45:60) to give **4** (6 mg).

Fraction D ($CHCl_3$:$Me_2CO$ 7:3, 50 g) was eluted with $CHCl_3$:MeOH (30:1, 20:1, and 10:1) yielding sub-fractions D1–D3. Sub-fraction D1 ($CHCl_3$:MeOH, 30:1, 20 g,) was fractionated by repeated CC, first on RP-18 with a gradient elution with MeOH:$H_2O$ (2:8 to 1:0) to yield fractions D1/1–D1/8. Subsequently, fraction D1/3 (2.27 g) was purified using a silica gel column ($CHCl_3$: MeOH, 50:1 to 10:1) to give sub-fractions D1/3/1–D1/3/8. Sub-fraction D1/3/3 was purified by PPLC (MeOH:$H_2O$, 40:60, 20 mL/min) to yield **5** (19 mg). Sub-fraction D2 (10 g, $CHCl_3$:MeOH, 20:1) was fractionated by repeated CC, first on RP-18 with gradient elution with MeOH:$H_2O$ (2:8 to 1:0) to yield fractions D2/1-D2/5. Subsequently, fraction D2/2 (0.87 g) was purified using a silica gel column ($CHCl_3$: isopropyl alcohol, 30:1 to 10:1) to afford sub-fractions D2/2/1 (180 mg), D2/2/2 (105 mg), D2/2/3 (150 mg), and D2/2/4 (120 mg). Sub-fraction D2/2/3 was purified by RP-18 CC (MeOH:$H_2O$, 40:60) to yield **2** (2 mg). Sub-fraction D3 (10:1 $CHCl_3$:MeOH, 6 g) was purified by CC on RP-18 (15:85-1:0, MeOH-$H_2O$) to yield fractions D3/1–D3/6. Subsequently, fraction D3/3 (1.26 g) was purified by CC on silica gel column (30:1-10:1 $CHCl_3$:isopropyl alcohol) to yield sub-fractions D3/3/1 (750 mg), D3/3/2 (85 mg), and D3/3/3 (120 mg). Compound **3** (6 mg) was precipitated from sub-fraction D3/3/1 by subsequent silica gel CC (20:1 $CHCl_3$:MeOH) and RP-18 (40:60 MeOH:$H_2O$).

**Laxiflorin S (1):** white, amorphous powder. $[\alpha]^{21.0}_{D}$ – 53.1 (*c* 0.10, MeOH). UV (MeOH) $\lambda_{max}$ (log $\varepsilon$): 199.2 (3.0), 237.2 (3.3) nm; IR (KBr) $\nu_{max}$ 3480, 2977, 2961, 2851, 1741, 1647, 1455, 1434, 1371, 1259, 1221, 1033 cm$^{-1}$; For $^1$H and $^{13}$C spectroscopic data, see Tables 1 and 2; EIMS: *m/z* 430 [M]$^+$; HREIMS [M]$^+$ *m/z* 430.2003 (calcd for $C_{24}H_{30}O_7$, 430.1992).

**Laxiflorin T (2):** white, amorphous powder. $[\alpha]^{24.5}_{D}$ + 1.2 (*c* 0.10, MeOH). UV (MeOH) $\lambda_{max}$ (log $\varepsilon$): 202.6 (2.7) nm; IR (KBr) $\nu_{max}$ 3521, 2958, 2933, 1740, 1726, 1696, 1428, 1379, 1285, 1116, 1040 cm$^{-1}$; For $^1$H and $^{13}$C spectroscopic data, see Tables 1 and 2; Positive ESIMS: m/z 471 [M + Na]$^+$; HREIMS [M]$^+$ *m/z* 448.2087 (calcd for $C_{24}H_{32}O_8$, 448.2097).

**Laxiflorin U (3):** white power. $[\alpha]^{22.0}_{D}$ + 21.6 (*c* 0.10, MeOH). UV (MeOH) $\lambda_{max}$ (log $\varepsilon$): 227 (3.03) nm. IR (KBr) $\nu_{max}$ 3402, 2961, 2924, 1780, 1723, 1700, 1671, 1630, 1379, 1228, 1072 cm$^{-1}$; For $^1$H and $^{13}$C spectroscopic data, see Tables 1 and 2; EIMS: m/z 334 [M]$^+$; HREIMS [M]$^+$ m/z 334.1416 (calcd for $C_{18}H_{22}O_6$, 334.1416).

**Laxiflorin V (4):** white, amorphous powder. $[\alpha]^{18.3}_{D}$ – 20.3 (*c* 0.10, MeOH). UV (MeOH) $\lambda_{max}$ (log $\varepsilon$): 203.0 (3.1) nm; IR

(KBr) $\nu_{max}$ 3440, 2926, 2867, 1708, 1690, 1641, 1459, 1449, 1411, 1378, 1307, 1237, 1052 $cm^{-1}$; For $^{1}H$ and $^{13}C$ spectroscopic data, see Tables 1 and 2; Positive ESIMS: *m/z* 343 $[M + Na]^+$; HREIMS $[M]^+$ *m/z* 320.2343 (calcd for $C_{20}H_{32}O_3$, 320.2351).

## Electronic Supplementary Material

Supplementary material is available in the online version of this article at http://dx.doi.org/10.1007/s13659-013-0057-0 and is accessible for authorized users.

## Acknowledgments

The authors are grateful to Prof. Xi-Wen Li of the Kunming Institute of Botany, Chinese Academy of Sciences, for the identification of the plant. This project was supported financially by the National Natural Science Foundation of China (No. 81172939), the West Light Foundation of the Chinese Academy of Sciences (J.X. Pu), the Major State Basic Research Development Program of China (No. 2009CB522300), the reservation talent project of Yunnan Province (2011CI043 to J.X. Pu), the Science and Technology Program of Yunnan Province (Nos. 2008IF010), and the Major Direction Projection Foundation of CAS Intellectual Innovation Project (No. KSCX2-EW-J-24 to J.X. Pu).

**Open Access** This article is distributed under the terms of the Creative Commons Attribution License which permits any use, distribution, and reproduction in any medium, provided the original author(s) and source are credited.

## Electronic supplementary material

Supplementary material, approximately 3.08 MB.
