# Supplementary material for: Four new diterpenoids from Isodon eriocalyx var. laxiflora
Source: Nat Prod Bioprospect. 2013 Aug 13;3(4):145–9. doi: 10.1007/s13659-013-0057-0 (PMC4131582; doi:10.1007/s13659-013-0057-0)

Electronic Supplementary Material

Four new diterpenoids from *Isodon eriocalyx* var. *laxiflora*

Wei-Guang WANG,[a,b] Xue DU,[a] Xiao-Nian LI,[a] Bing-Chao YAN,[a,b] Min ZHOU,[a,b] Hai-Yan WU,[a,b] Rui ZHAN,[a,b] Ke DONG,[a] Jian-Xin PU,[a,]* and Han-Dong SUN[a,]*

[a]State Key Laboratory of Phytochemistry and Plant Resources in West China, Kunming Institute of Botany, Chinese Academy of Sciences, Kunming 650201, China

[b]University of Chinese Academy of Sciences, Beijing 100049, China

Received 18 July 2013; Accepted 2 August 2013

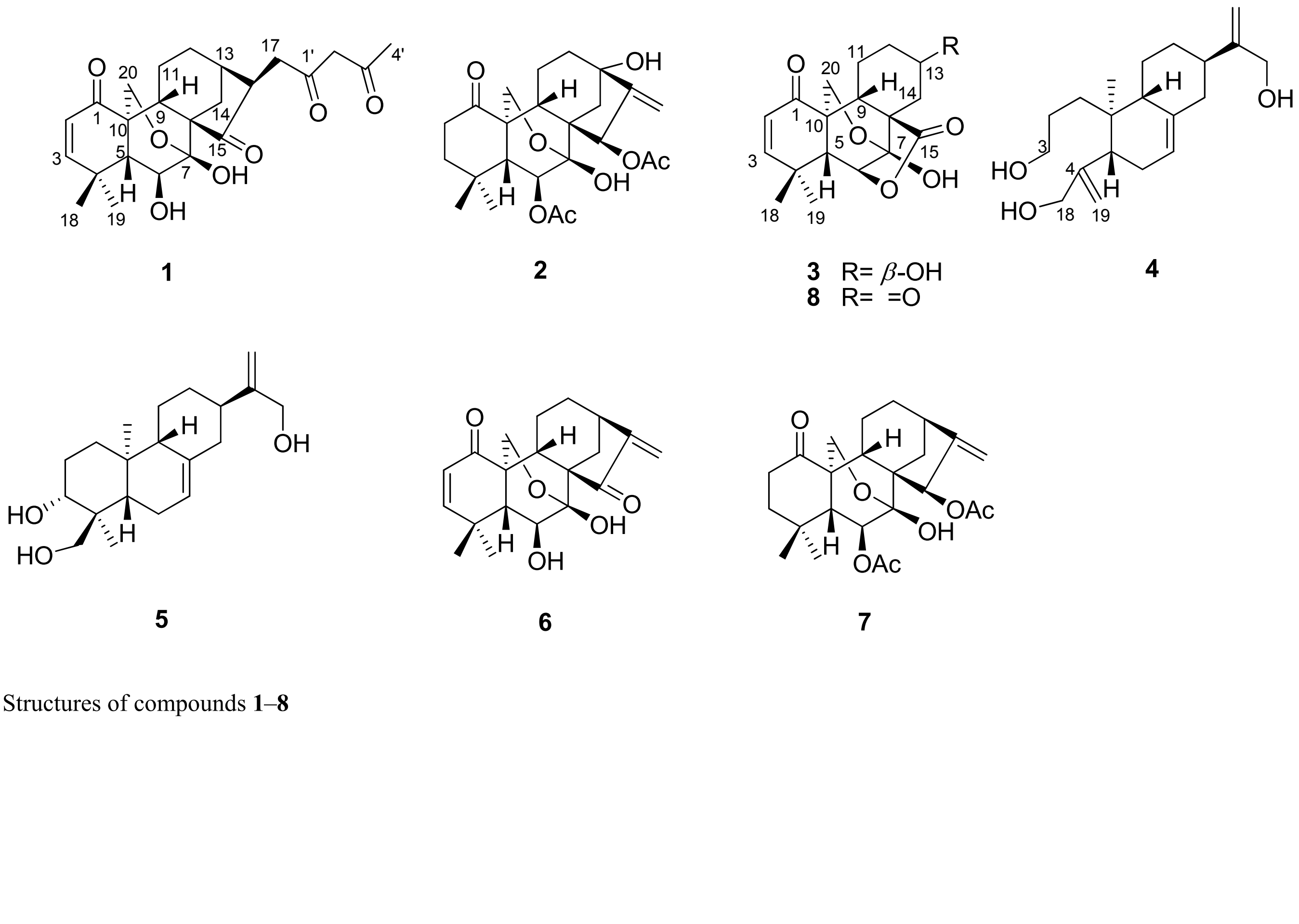

Structures of compounds **1**–**8**

*To whom correspondence should be addressed. E-mail: pujianxin@mail.kib.ac.cn (J.X. Pu); hdsun@mail.kib.ac.cn (H.D. Sun)

Contents of Supporting Information

No.	Contents:	Pages:
1.	**Figures S1-S7**. HREI MS, ^{1}H NMR, ^{13}C NMR, HSQC, ^{1}H-^{1}H COSY, HMBC, and ROESY spectra of **1**	3-9
2.	**Figures S8-S10**. HREI MS, ^{1}H NMR, and ^{13}C NMR spectra of **2**	10-12
3.	**Figures S11-S13**. HREI MS, ^{1}H NMR, and ^{13}C NMR spectra of **3**	13-15
4.	**Figures S14-S20**. HREI MS, ^{1}H NMR, ^{13}C NMR, HSQC, ^{1}H-^{1}H COSY, HMBC, and ROESY spectra of **4**	16-22

Figures S1 HRESI MS spectrum of **1**

Single Mass Analysis

Tolerance = 10.0 PPM / DBE: min = -10.0, max = 120.0

Selected filters: None

Monoisotopic Mass, Odd and Even Electron Ions

18 formula(e) evaluated with 1 results within limits (up to 51 closest results for each mass)

Elements Used:

C: 0-200 H: 0-400 O: 6-8

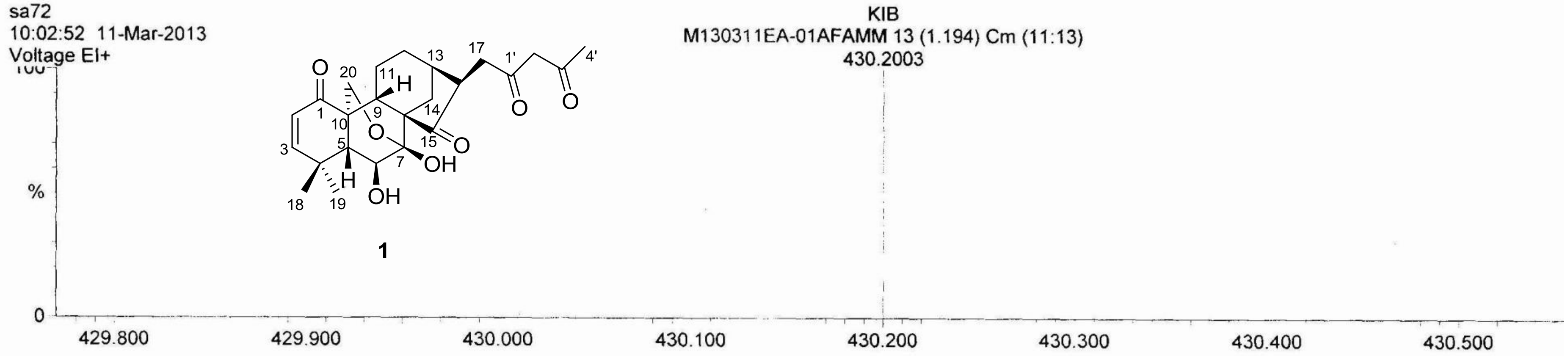

Minimum:				-10.0		
Maximum:		200.0	10.0	120.0		
Mass	Calc. Mass	mDa	PPM	DBE	i-FIT	Formula
430.2003	430.1992	1.1	2.6	10.0	5546071.5	C24 H30 O7

Figures S2 ^{1}H NMR spectrum of **1**

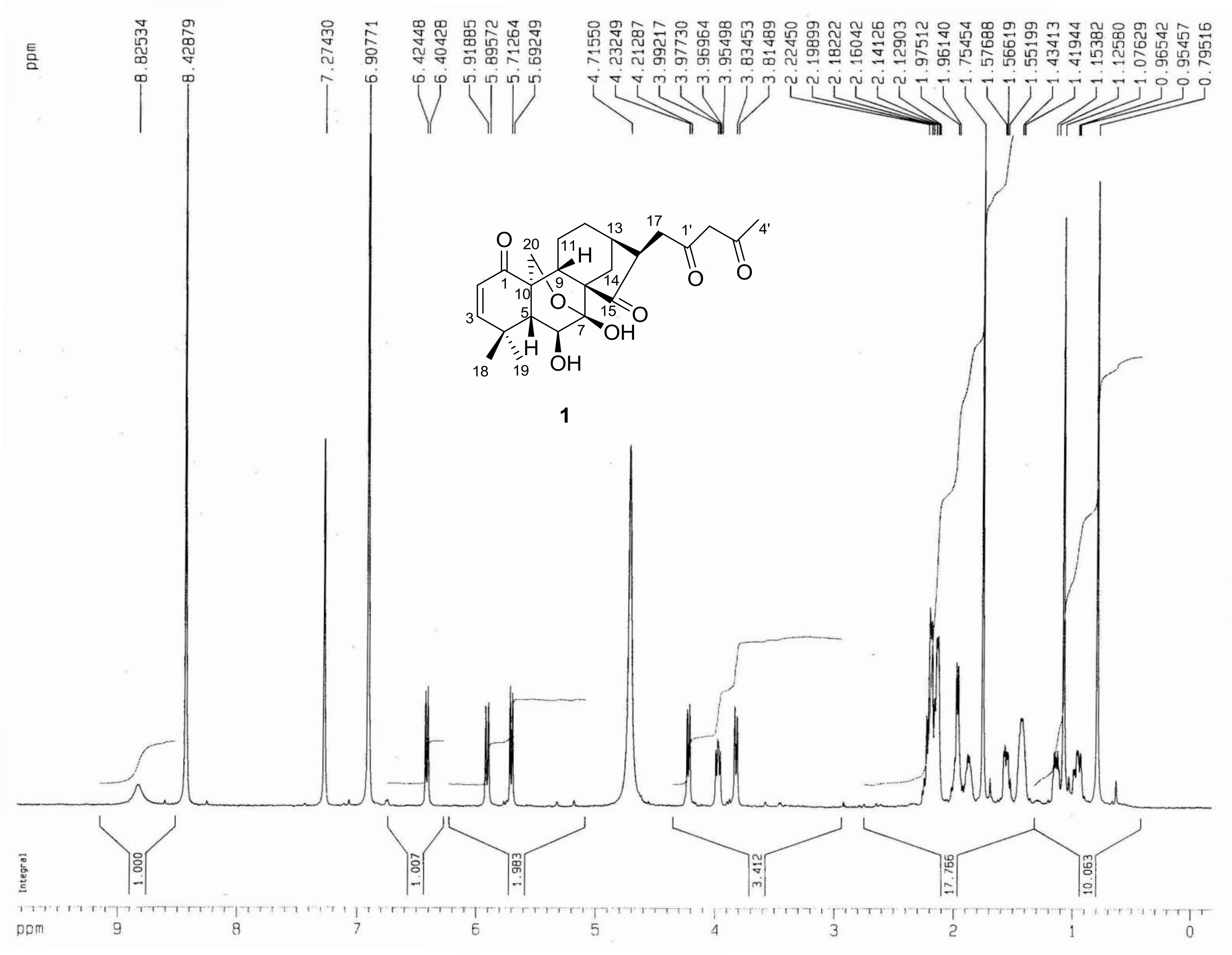

Figure S3. ^{13}C NMR spectrum of **1**

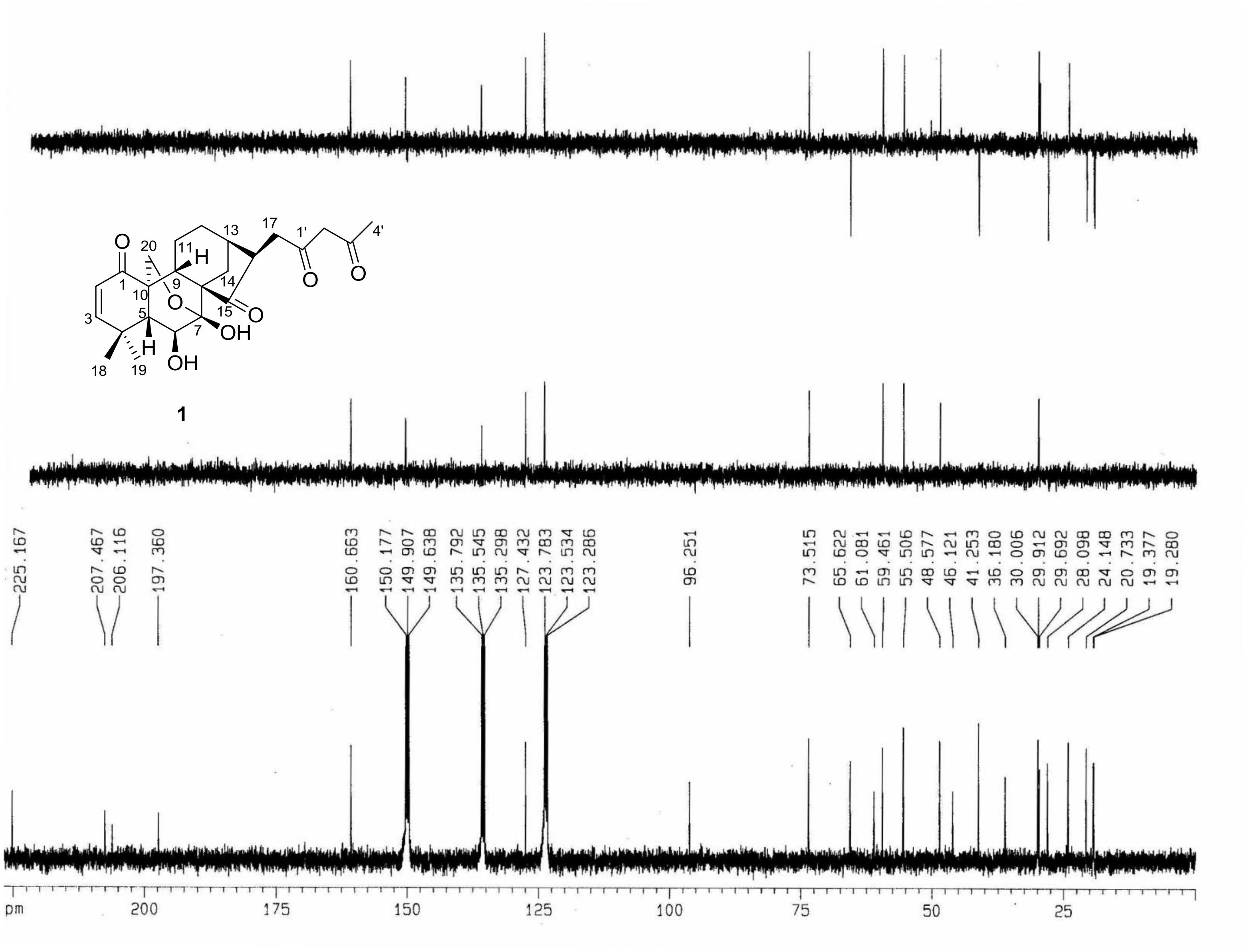

Figure S4. HSQC spectrum of **1**

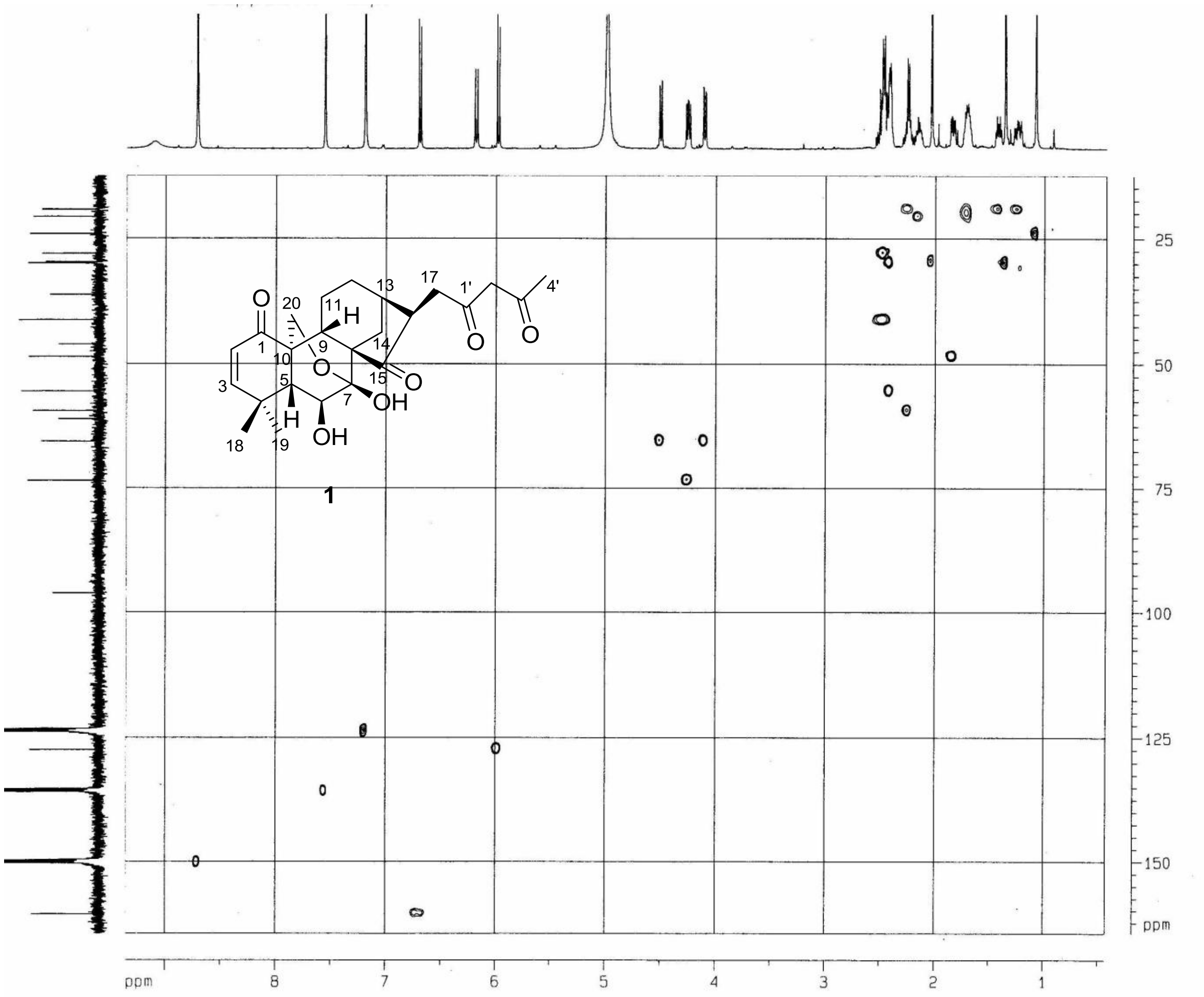

Figure S5 ^{1}H-^{1}H COSY spectrum of **1**

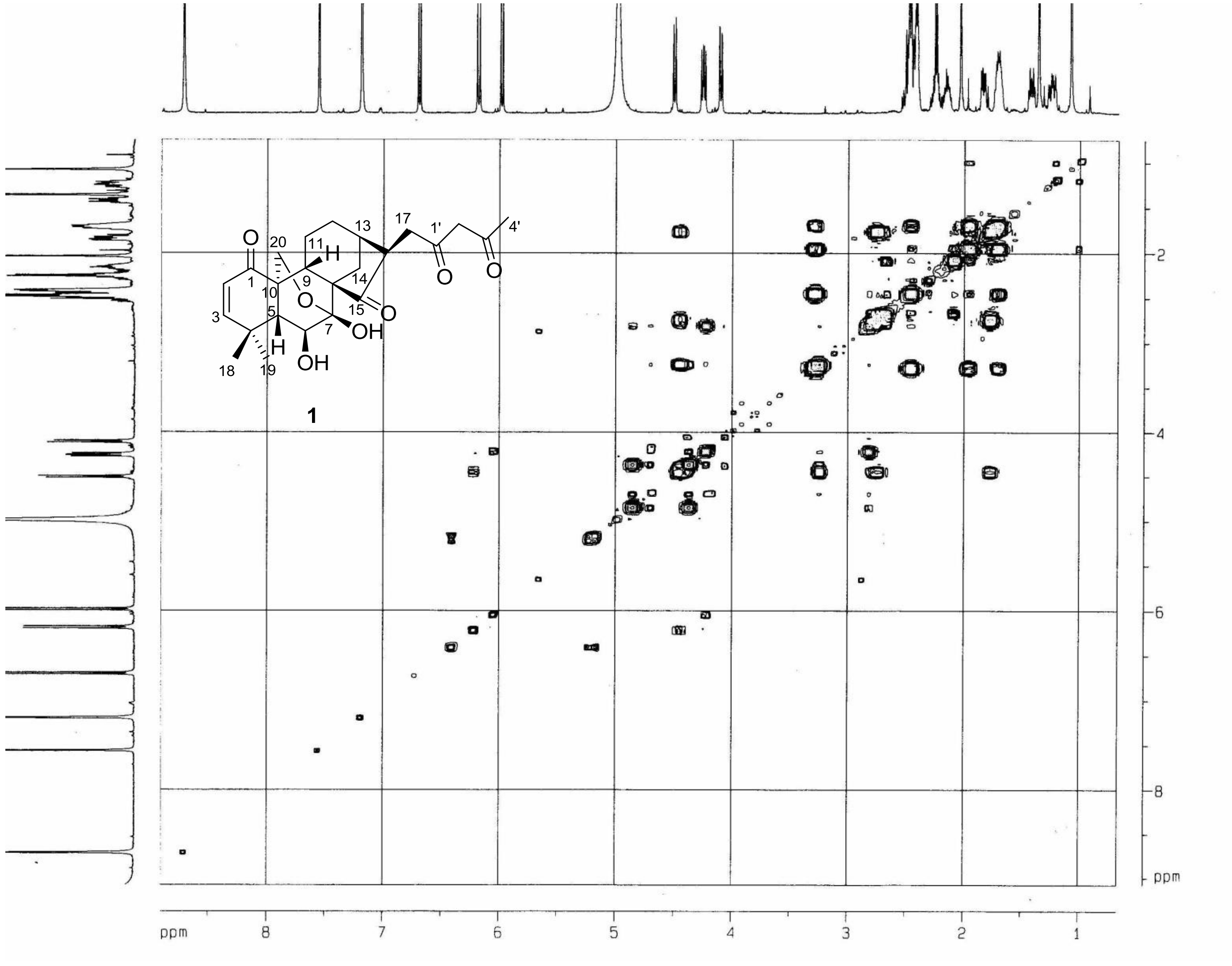

Figure S6 HMBC spectrum of **1**

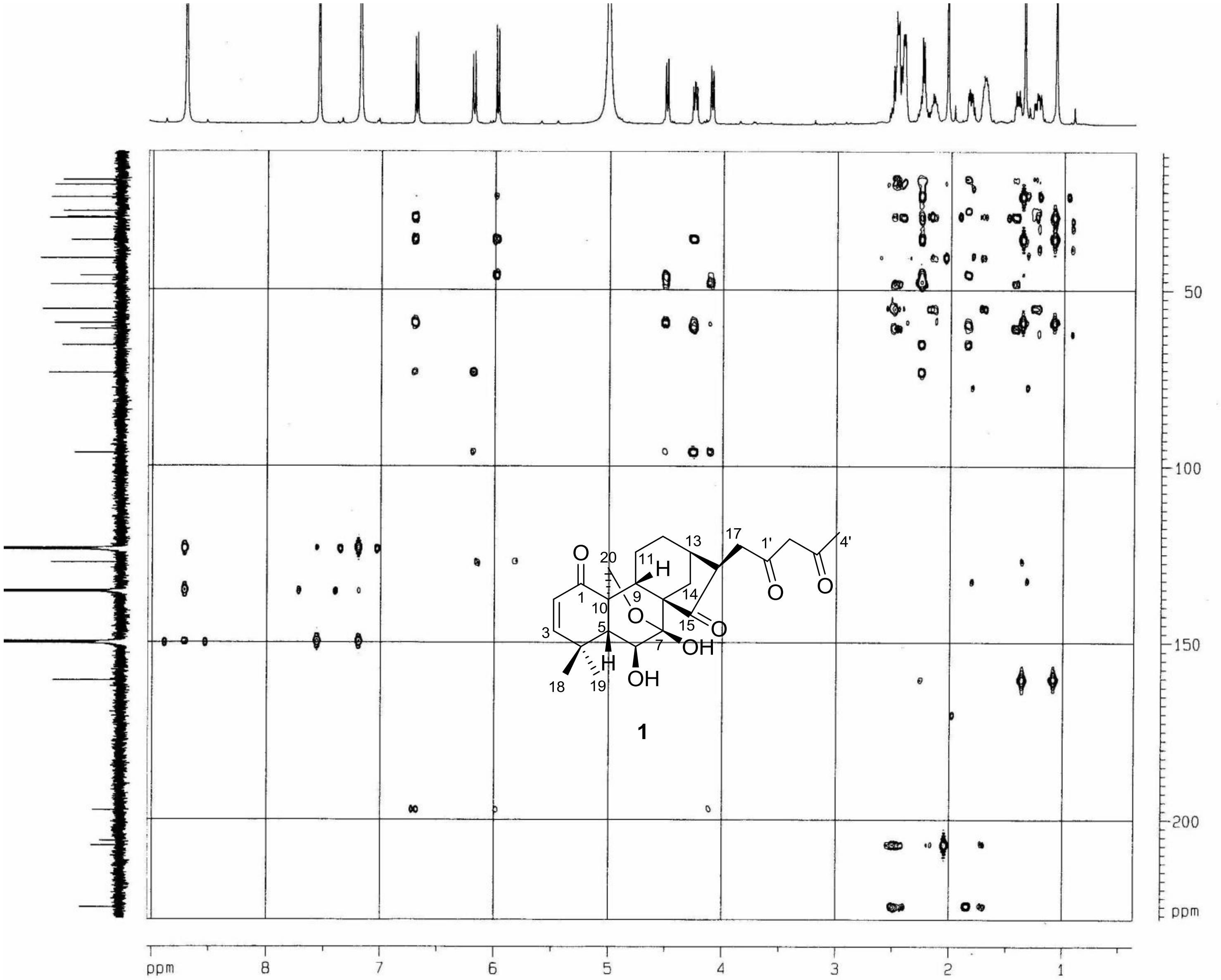

Figure S7 ROESY spectrum of **1**

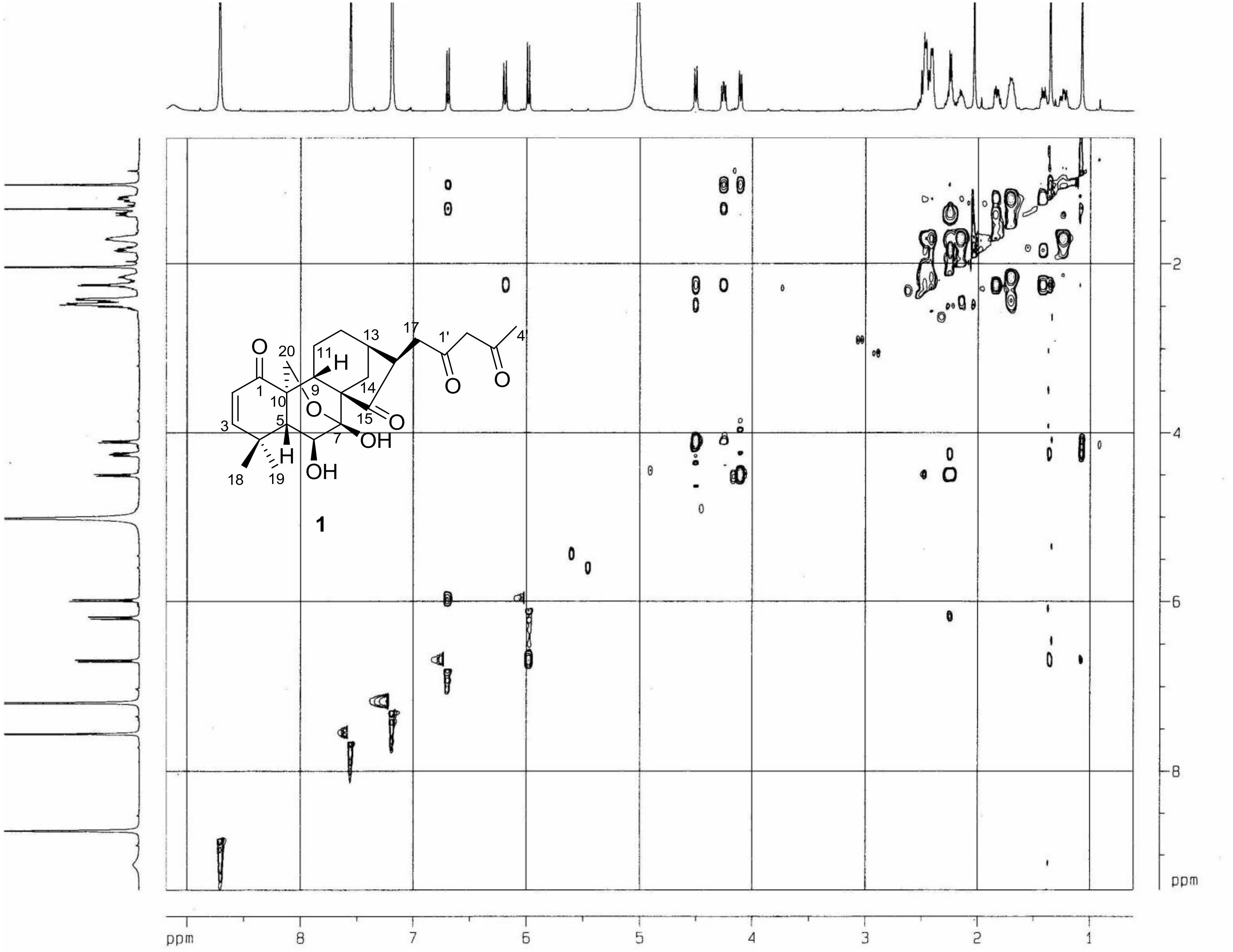

Figures S8 HRESI MS spectrum of **2**

Single Mass Analysis

Tolerance = 10.0 PPM / DBE: min = -10.0, max = 120.0

Selected filters: None

Monoisotopic Mass, Odd and Even Electron Ions

19 formula(e) evaluated with 1 results within limits (up to 51 closest results for each mass)

Elements Used:

C: 0-200 H: 0-400 O: 6-8

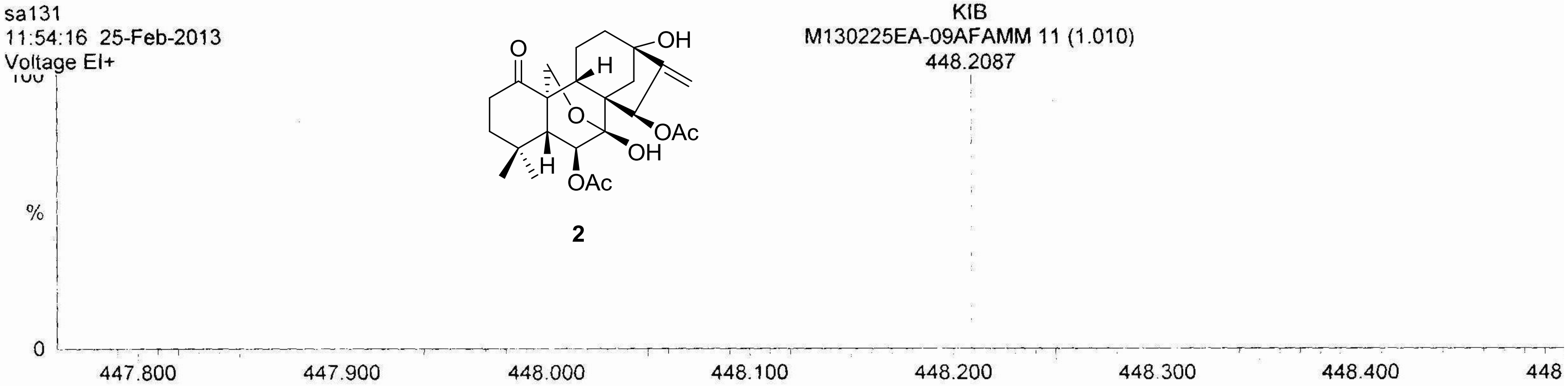

Minimum:				-10.0		
Maximum:		200.0	10.0	120.0		
Mass	Calc. Mass	mDa	PPM	DBE	i-FIT	Formula
448.2087	448.2097	-1.0	-2.2	9.0	5546027.0	C24 H32 O8

Figures S9 ^{1}H NMR spectrum of **2**

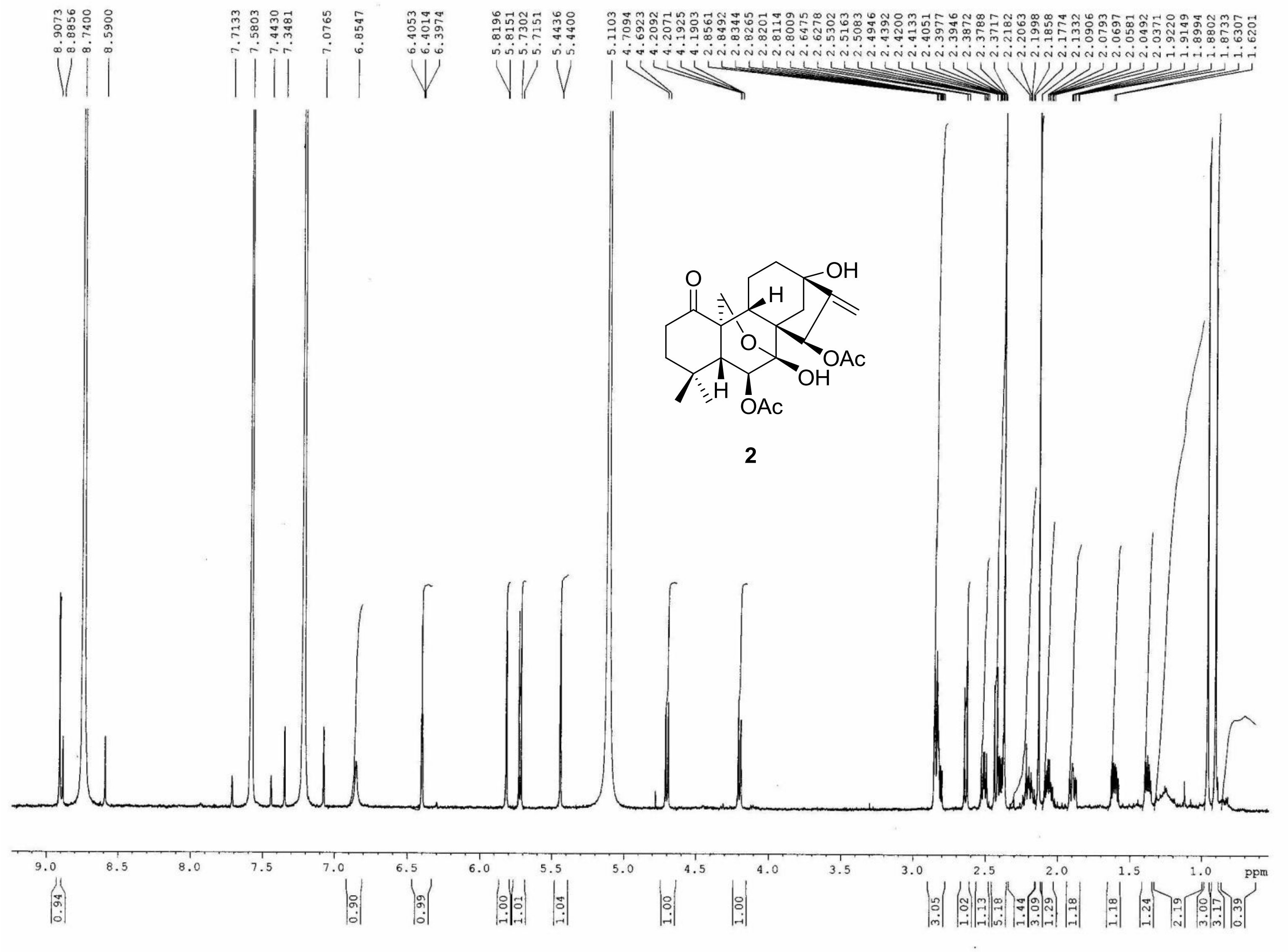

Figure S10. ^{13}C NMR spectrum of **2**

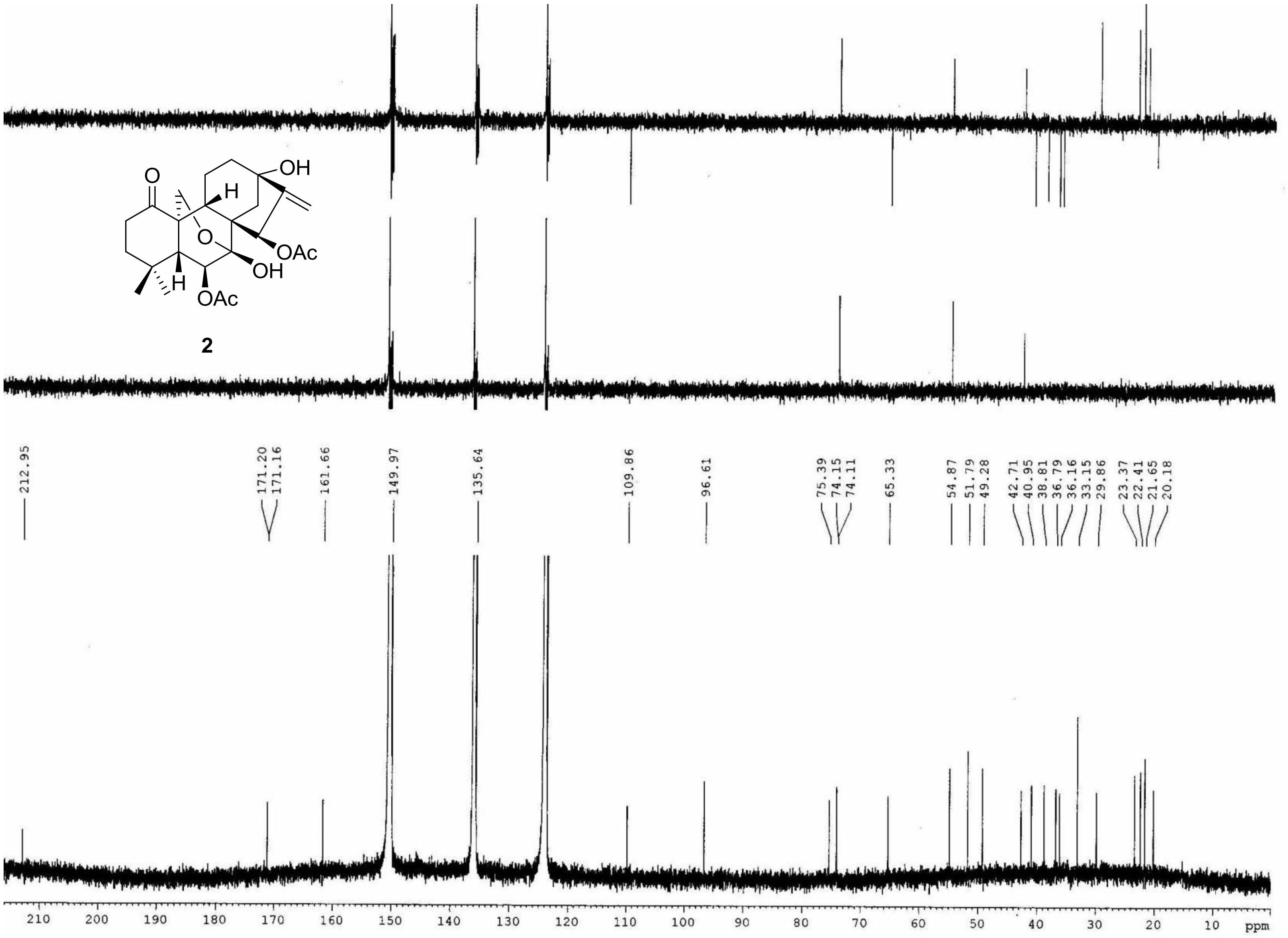

Figures S11 HRESI MS spectrum of **3**

Monoisotopic Mass, Odd and Even Electron Ions
15 formula(e) evaluated with 1 results within limits (up to 51 closest results for each mass)
Elements Used:
C: 0-200 H: 0-400 O: 6-8

sapple230
09:51:23 11-Mar-2013
Voltage EI+

KIB
M130311EA-03AFAMM 24 (2.204)

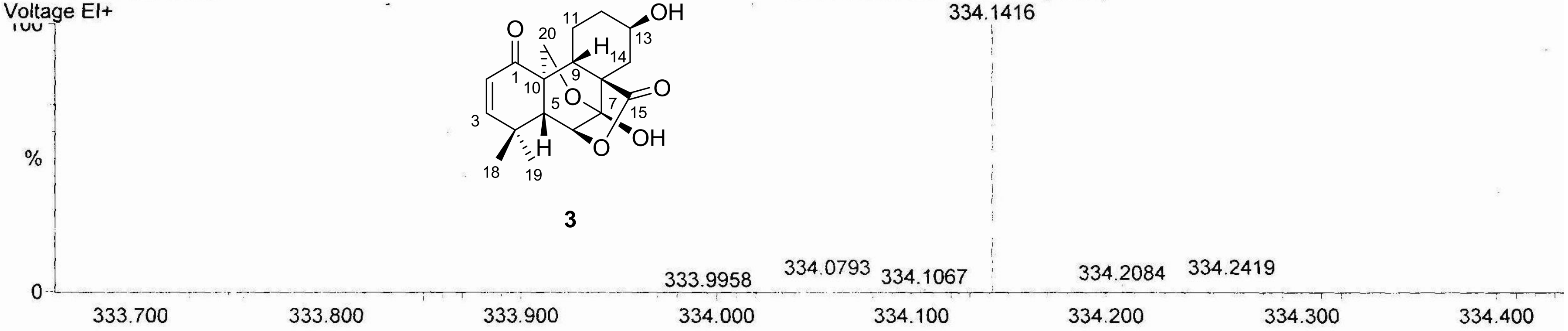

Minimum:				-10.0		
Maximum:		200.0	10.0	120.0		
Mass	Calc. Mass	mDa	PPM	DBE	i-FIT	Formula
334.1416	334.1416	0.0	0.0	8.0	5546157.5	C18 H22 O6

Figures S12 ^{1}H NMR spectrum of **3**

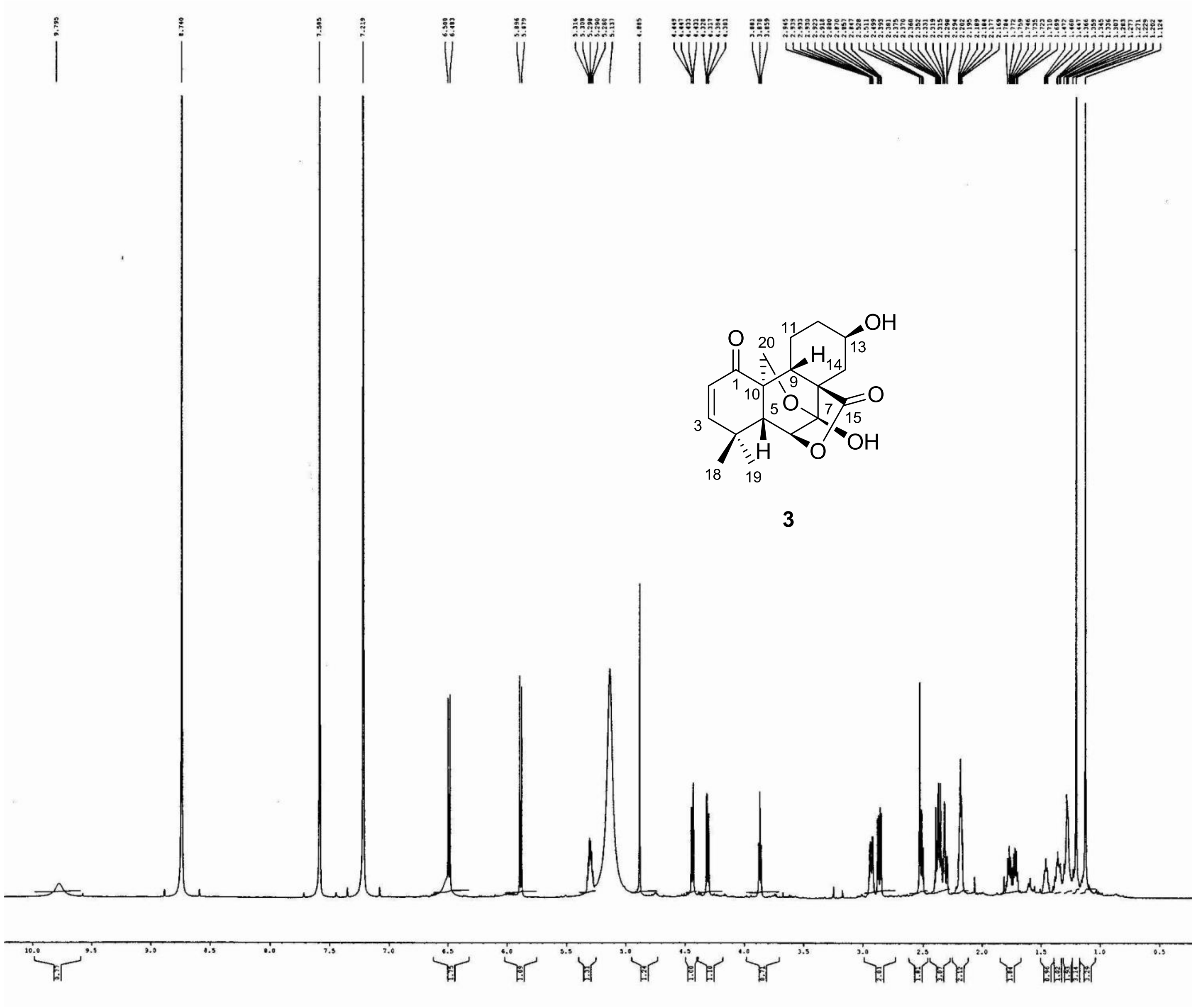

Figure S13. ^{13}C NMR spectrum of **3**

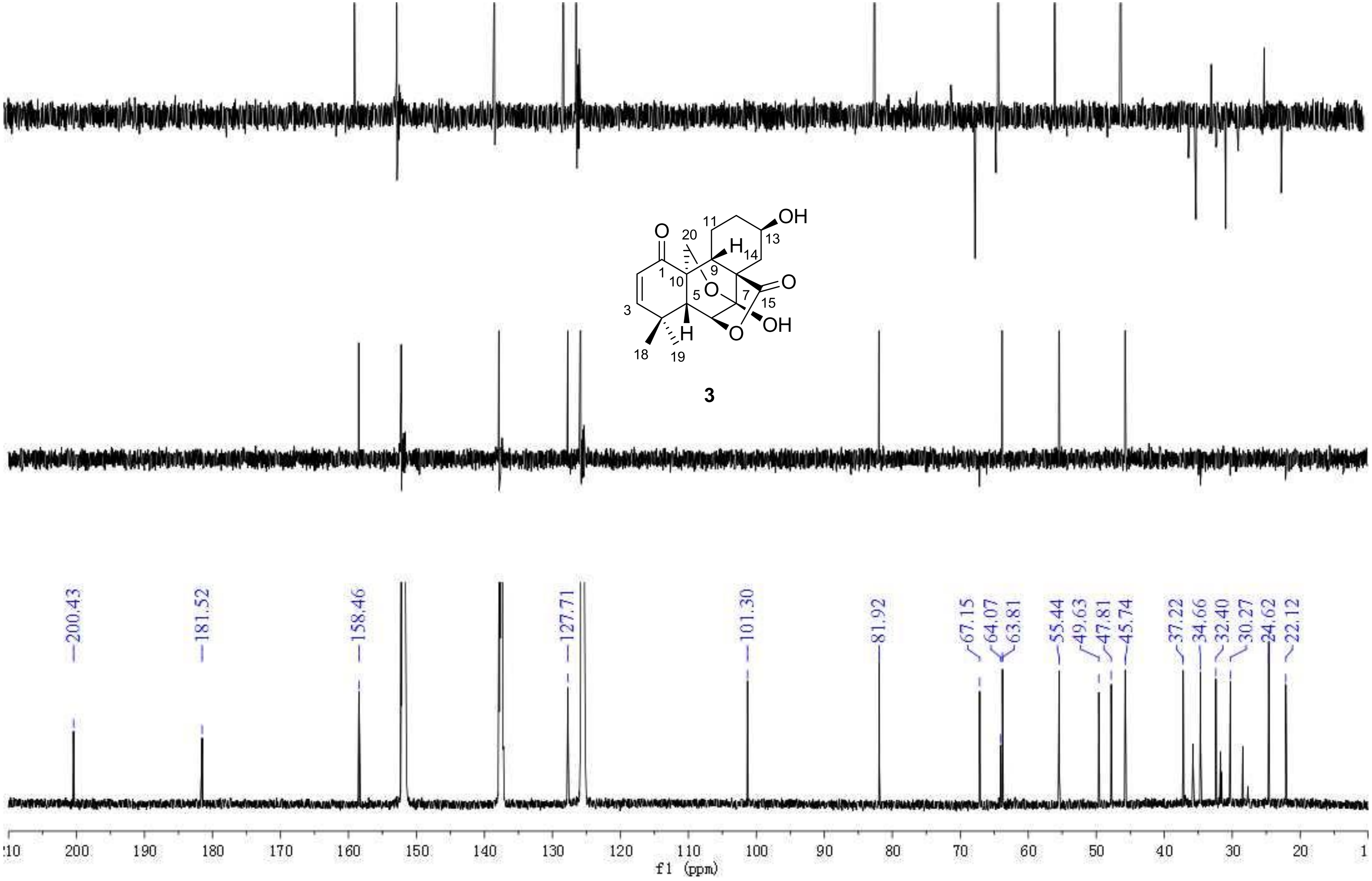

Figure S14. HREI spectrum of **4**

Single Mass Analysis

Tolerance = 10.0 PPM / DBE: min = -10.0, max = 120.0
Selected filters: None

Monoisotopic Mass, Odd and Even Electron Ions
16 formula(e) evaluated with 1 results within limits (up to 51 closest results for each mass)
Elements Used:
C: 0-200 H: 0-400 O: 2-4

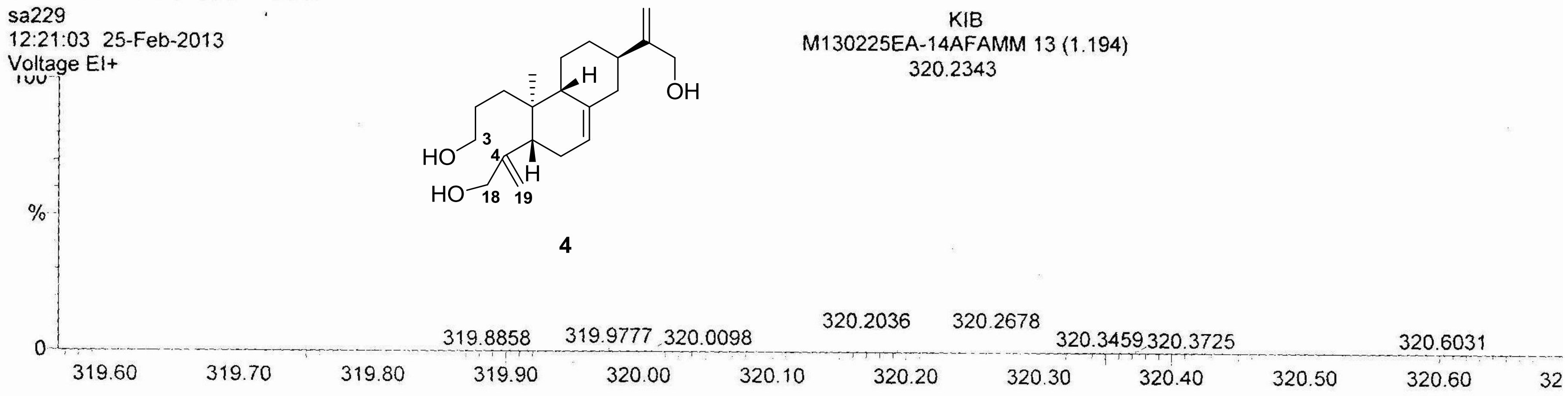

Minimum:				-10.0		
Maximum:		200.0	10.0	120.0		

Mass	Calc. Mass	mDa	PPM	DBE	i-FIT	Formula
320.2343	320.2351	-0.8	-2.5	5.0	5546141.0	C20 H32 O3

Figure S15. ^{1}H NMR spectrum of **4**

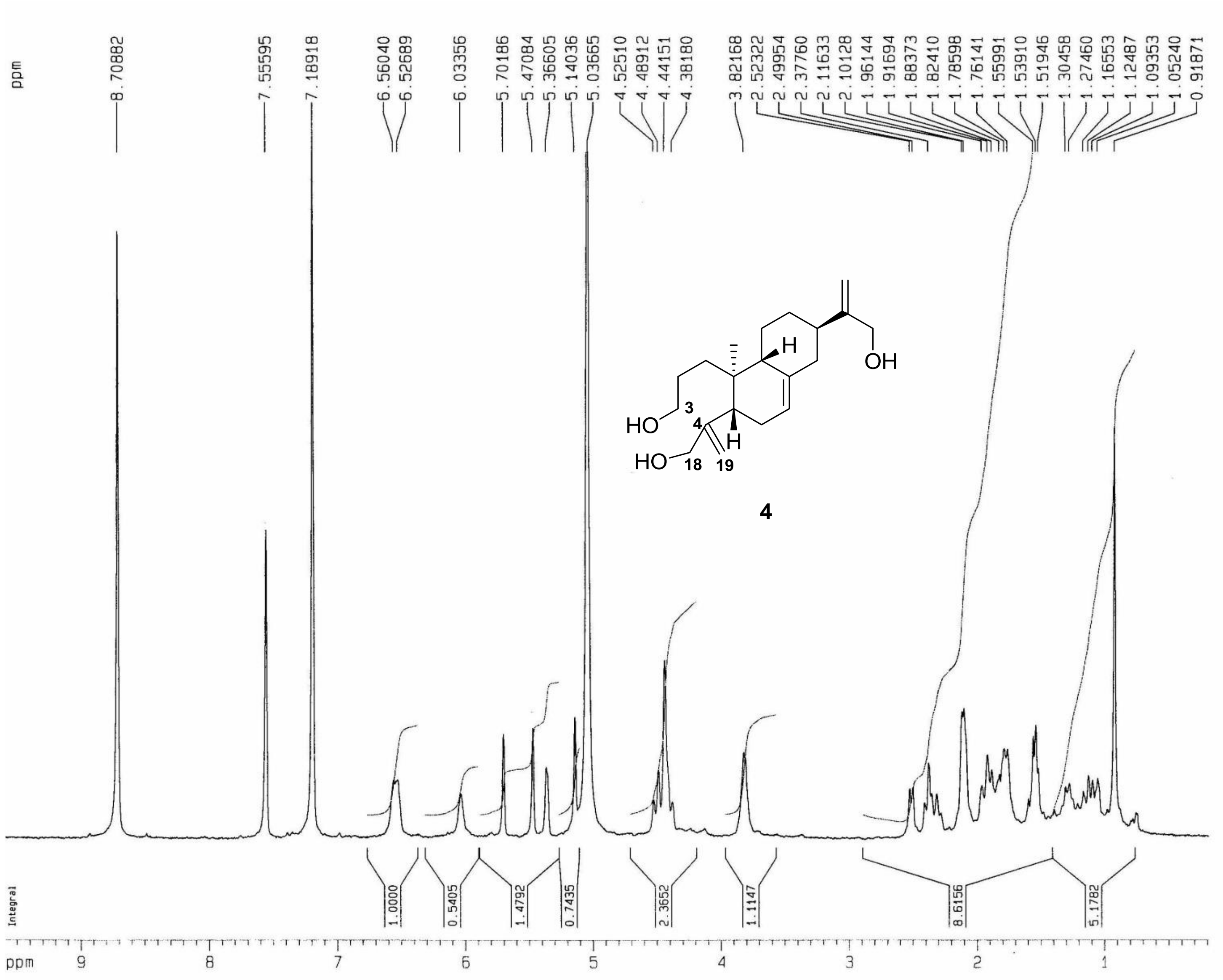

Figure S16. ^{13}C NMR spectrum of **4**

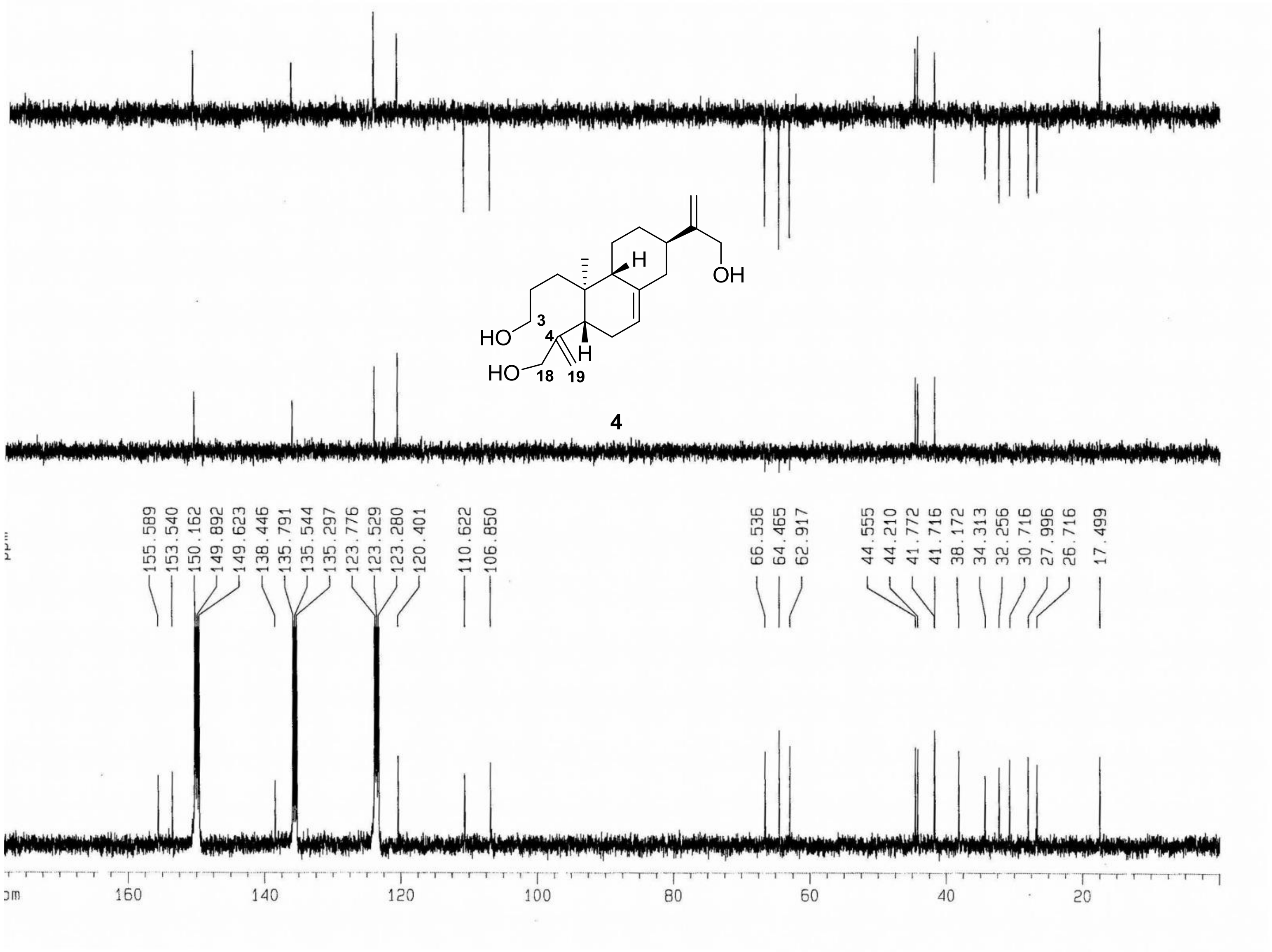

Figure S17. HSQC spectrum of **4**

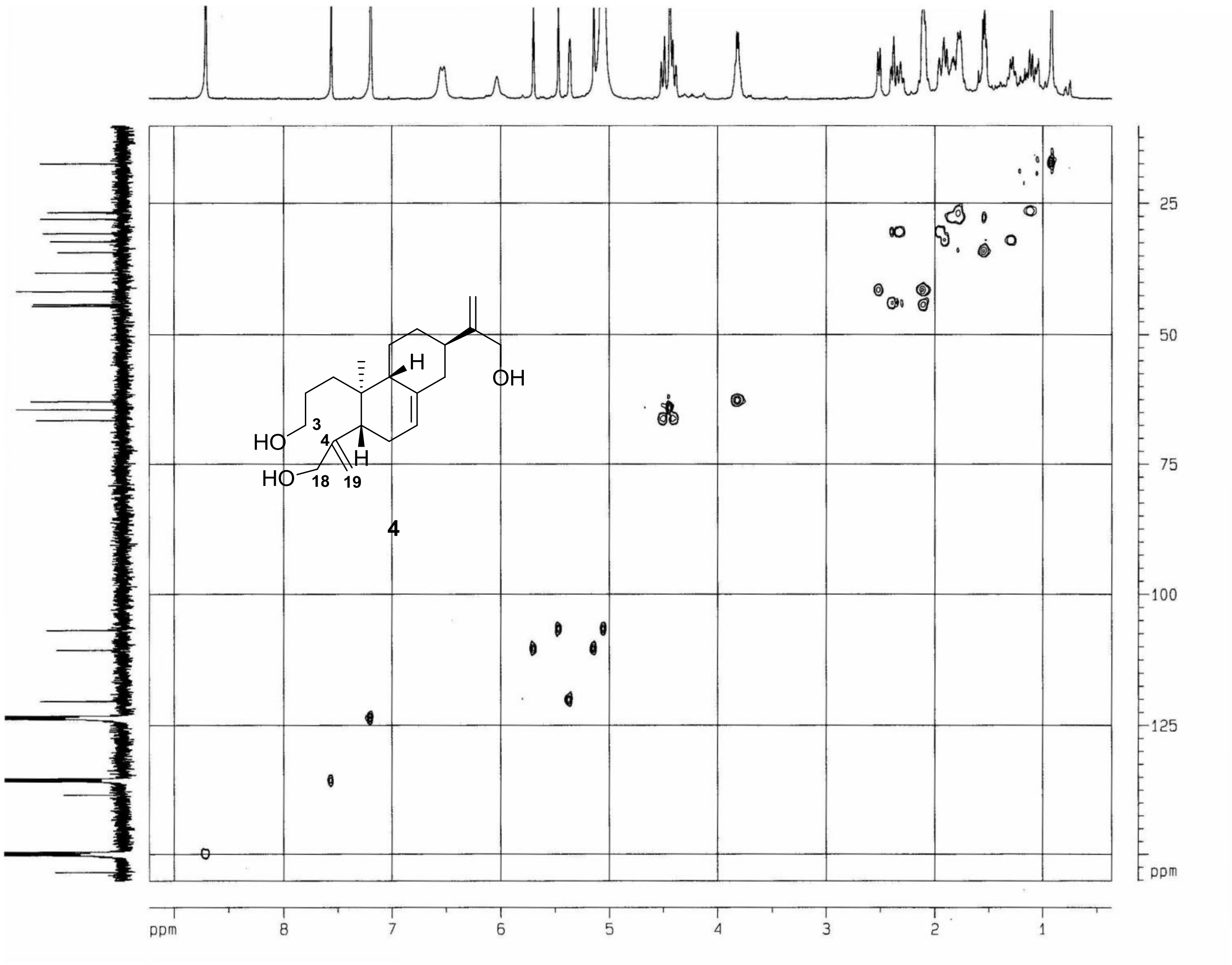

Figure S18. ^{1}H-^{1}H COSY spectrum of **4**

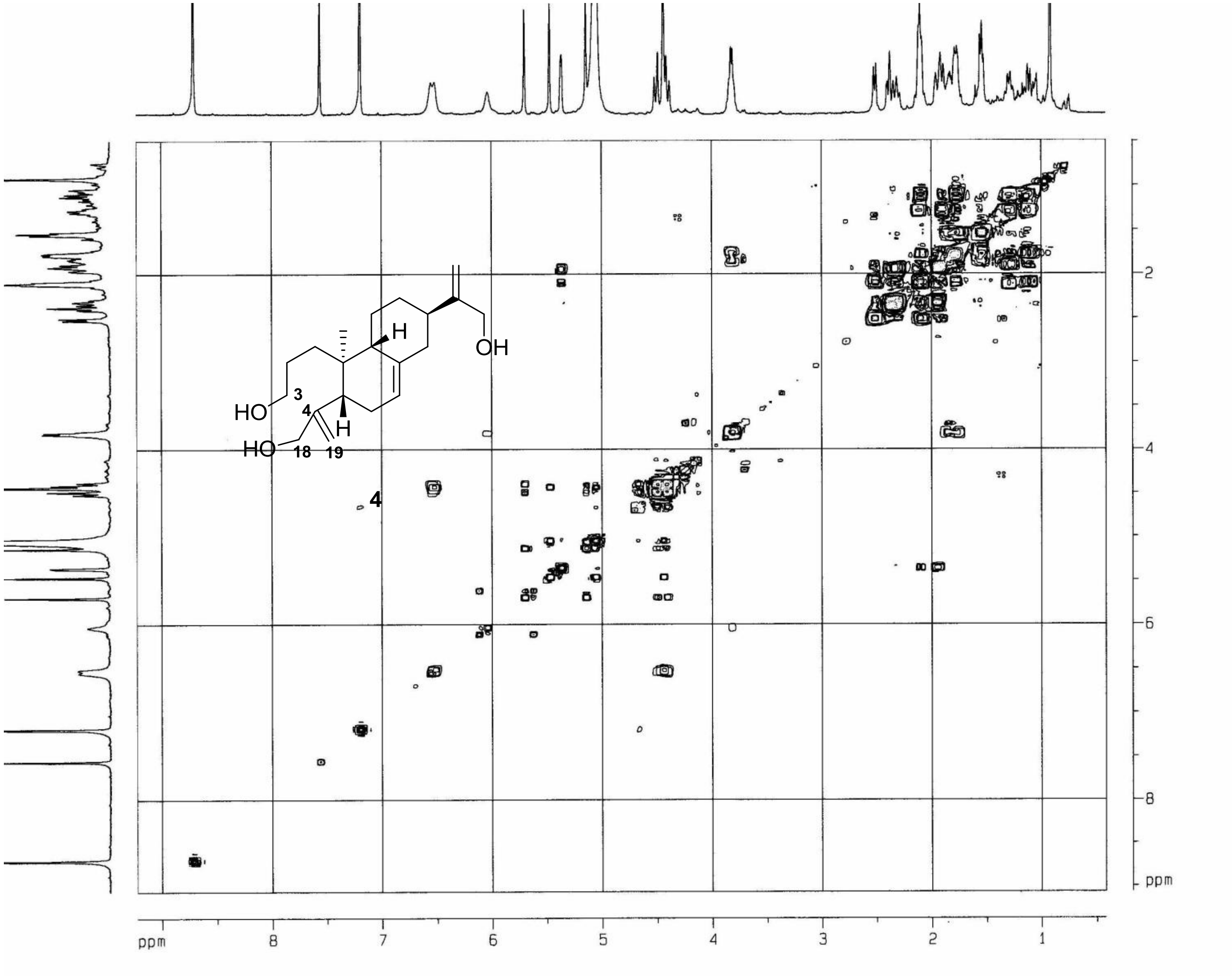

Figure S19. HMBC spectrum of **4**

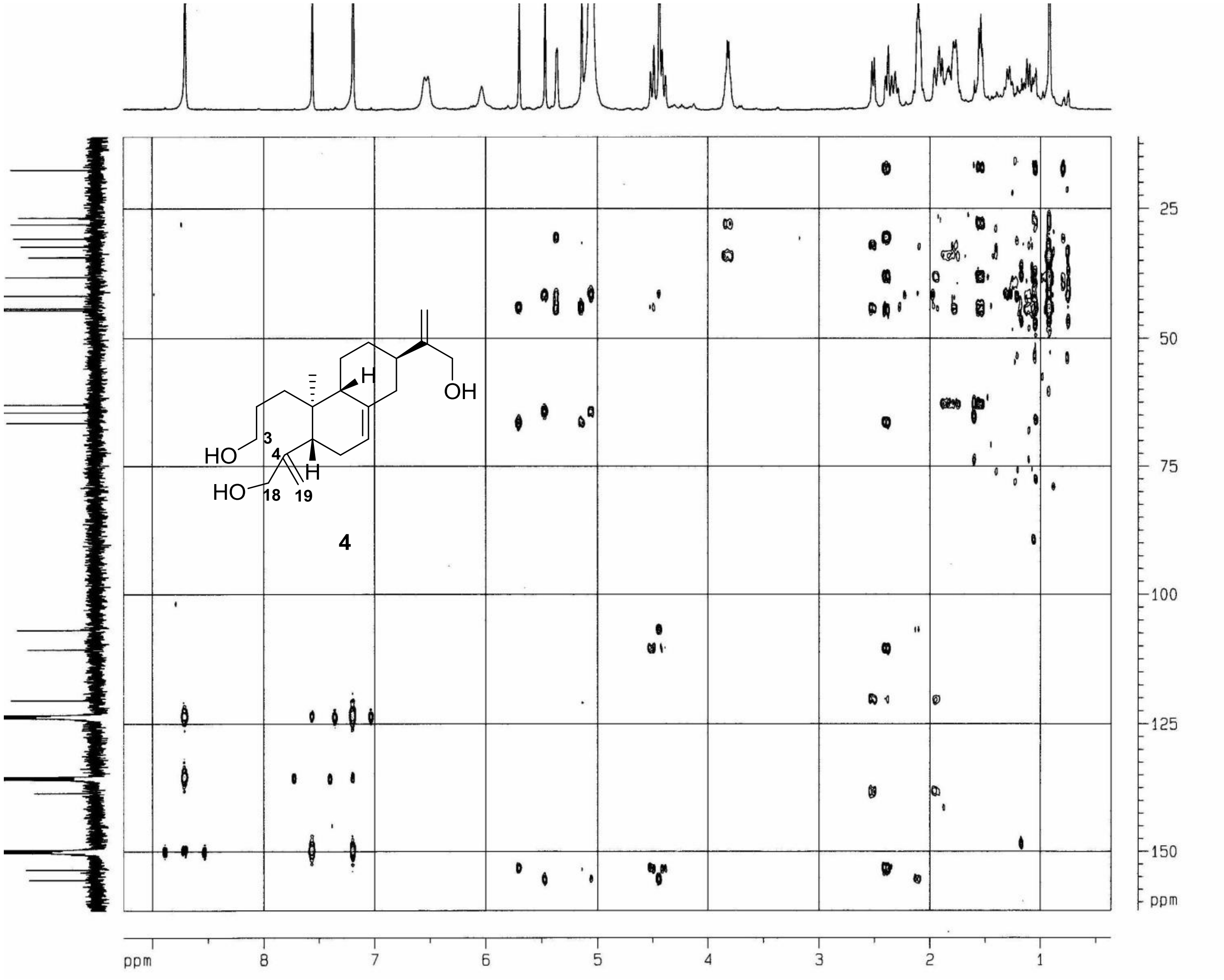

Figure S20. ROESY spectrum of **4**

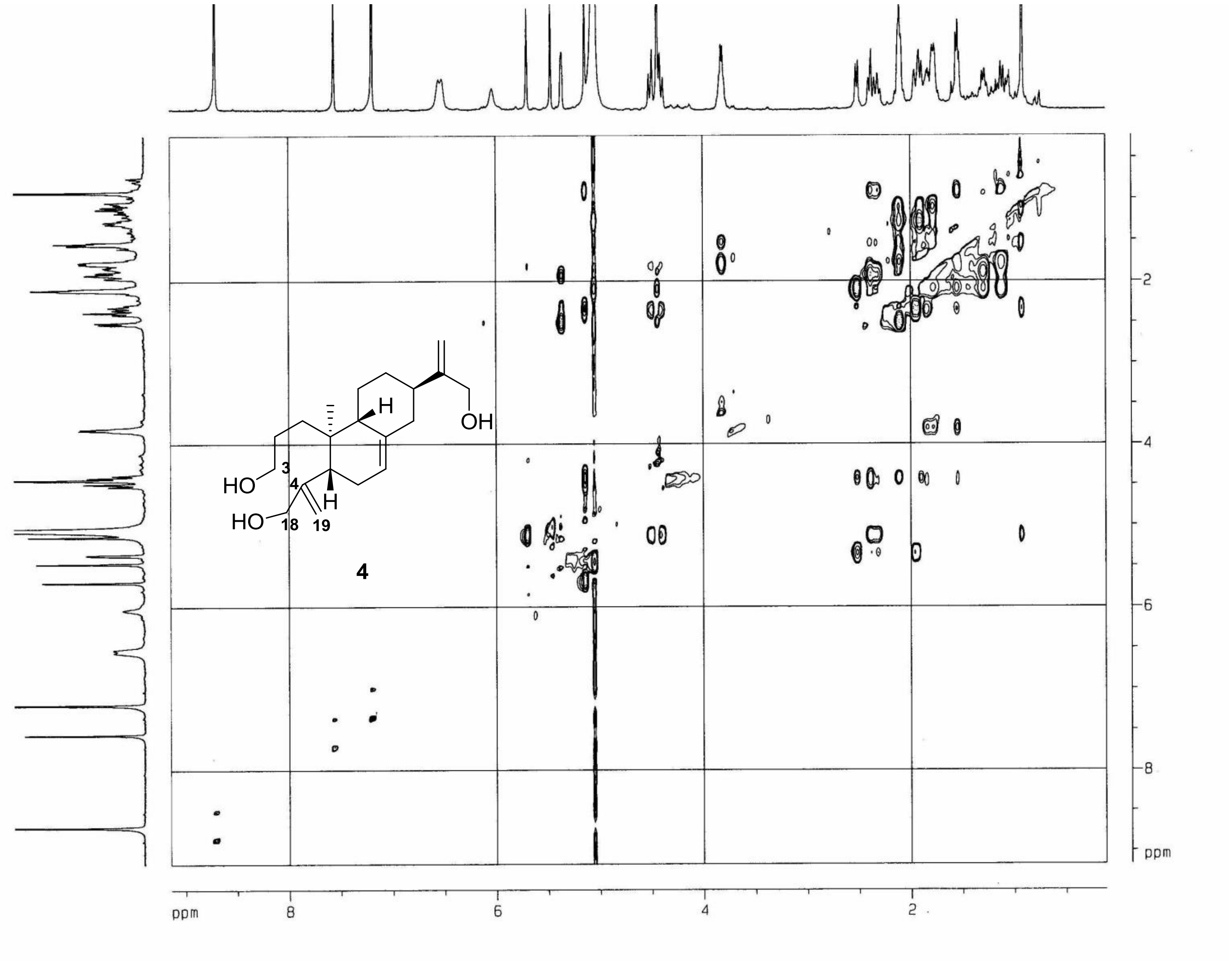

Supplement: Supplementary file 1 — Supplementary material, approximately 3.08 MB. [file 13659_2013_57_MOESM1_ESM.pdf]
